# Structural, functional and blood perfusion changes in the rat retina associated with elevated intraocular pressure, measured simultaneously with a combined OCT+ERG system

**DOI:** 10.1371/journal.pone.0193592

**Published:** 2018-03-06

**Authors:** Bingyao Tan, Benjamin MacLellan, Erik Mason, Kostadinka Bizheva

**Affiliations:** 1 Department of Physics and Astronomy, University of Waterloo, Waterloo, Ontario, Canada; 2 School of Optometry and Vision Science, University of Waterloo, Waterloo, Ontario, Canada; 3 Department of System Design Engineering, University of Waterloo, Waterloo, Ontario, Canada; Massachusetts Eye & Ear Infirmary, Harvard Medical School, UNITED STATES

## Abstract

Acute elevation of intraocular pressure (IOP) to ischemic and non-ischemic levels can cause temporary or permanent changes in the retinal morphology, function and blood flow/blood perfusion. Previously, such changes in the retina were assessed separately with different methods in clinical studies and animal models. In this study, we used a combined OCT+ ERG system in combination with Doppler OCT and OCT angiography (OCTA) imaging protocols, in order to evaluate simultaneously and correlate changes in the retinal morphology, the retinal functional response to visual stimulation, and the retinal blood flow/blood perfusion, associated with IOP elevation to ischemic and non-ischemic levels in rats. Results from this study suggest that the inner retina responds faster to IOP elevation to levels greater than 30 mmHg with significant reduction of the total retinal blood flow (TRBF), decrease of the capillaries’ perfusion and reduction of the ON bipolar cells contribution to the ERG traces. Furthermore, this study showed that ischemic levels of IOP elevation cause an additional significant decrease in the ERG photoreceptor response in the posterior retina. Thirty minutes after IOP normalization, retinal morphology, blood flow and blood perfusion recovered to baseline values, while retinal function did not recover completely.

## Introduction

Elevated intraocular pressure (IOP) is a well-known risk factor in open angle glaucoma (OAG) and therefore it is the most studied pathogenic characteristic of OAG. In the past, multiple studies have reported separately the effect of IOP elevation on retinal blood flow or retinal function. Blood flow detection methods included laser Doppler flowmetry[1,2], ultrasound[3], magnetic resonance imaging[4], adaptive optics optical coherence tomography (OCT)[5] and adaptive optics scanning laser ophthalmology[6]. Visually evoked changes in the retinal function were typically assessed with electroretinography (ERG) or visual evoked potential[7]. Previous studies on animal models of elevated IOP-induced changes in the retinal blood flow and retinal function showed large variability of results and conclusions within and between different studies, most likely due to use of different imaging systems or focusing on different animal related parameters such as blood pressure[8], animal age[9,10] and strain[11]. To date, only a few studies have reported assessment of both retinal blood flow and function during IOP manipulation[12–15]. In all of those studies, either separate groups of animals were used, or ocular blood flow (combined retinal and choroidal flow) was measured invasively by a laser Doppler flowmetry probe. A combined OCT and ERG system would enable simultaneous, non-invasive imaging and assessment of retinal morphology, blood flow and function, which would reduce the variance in the acquired data. Such a combined OCT+ERG system has been used recently to assess simultaneously and correlate visually evoked intrinsic optical signals with ERG recordings and with retinal morphology in chicken[16,17]. The same system was also used to assess neurovascular coupling in the rat retina by measuring simultaneously and correlating visually evoked changes in the retinal blood flow and function[18]. Therefore, the OCT+ERG technology combined with various scanning protocols for Doppler OCT and OCT angiography (OCTA) could serve as a very useful research tool to investigate the dynamic relationship between morphological, blood flow and functional changes in the retina for various retinal diseases.

In animal studies, both acute and chronic IOP elevations are well-documented to cause both reduction in the retinal blood flow and decrease in the retinal functional response to visual stimulation. Specifically, in rodents[19,20], acute IOP elevation is associated with temporary impaired retinal cell function, assessed by ERG, for IOP levels of 50 mmHg and higher, while in other studies, reduced retinal blood flow[3,21–23] was detected at IOP levels as low as 30 mmHg. The difference in the ways retinal blood flow and function are resistant to changes in their responses to acute IOP elevation was partially explained by a mathematical model[12] which stated that increased oxygen extraction compensates for the relative ischemia caused by the reduced blood flow in order to sustain retinal function. This model was validated in studies on rodents with normal and abnormal blood pressure[24] and with/without diabetes[15] where the IOP was elevated using anterior chamber cannulation. In those studies, all animals exhibited more preserved retinal function compared to retinal blood flow for IOP elevation to moderate levels, while both attenuated retinal function and blood flow were observed for ischemic IOP levels (>60 mmHg).

Recent studies[11,25,26] by our research group also examined changes in the rat retinal function in response to IOP elevation in different rat strains and under different types of anesthesia. The IOP in those studies was elevated to a non-ischemic level of 35 mmHg using a vascular loop[27]. In one of the studies, the IOP was also raised to an ischemic level of 80 mmHg using the same method. Results from all of those studies showed temporary increase in the ERG a-wave and b-wave magnitudes by ~ 5x to 7x (supra normal ERG) during IOP elevation to 35 mmHg, compared to baseline and post-loop measurements, while no significant ERG response was observed for IOP elevation to 80 mmHg. Given the close relationship between retinal function and retinal blood flow, it is important to determine the retinal blood flow response to acute IOP elevation induced by the vascular loop, as well as to correlate the retinal blood flow and functional changes for different levels of IOP spanning from non-ischemic to ischemic levels. Therefore, in this study, we utilized a combined OCT+ERG system, as well as Doppler OCT and OCTA image acquisition protocols, to measure simultaneously changes in the rat retina structure, blood flow, blood perfusion and physiological function, in response to step-wise IOP elevation from 10 mmHg (normal level for healthy rat retina) to 70 mmHg (ischemic level).

## Materials and methods

### OCT+ERG system and data acquisition

The combined OCT+ERG system used for this study was developed recently by our research group for investigating the neurovascular coupling in the rat retina[17,18,28] and the effect of acutely elevated IOP on the retinal morphology and function. Briefly, the OCT system operates in the 1060 nm spectral region and provides ~3.5 μm axial and ~5 μm lateral resolution in retinal tissue with ~100 dB SNR for 1.7 mW optical power of the imaging beam incident on the cornea. An intensity based OCTA protocol[21] was used to generate angiographic images of the retina around the optic nerve head (ONH) by calculating the decorrelation between repeated cross-sectional images acquired at the same position in the retina (3.4 mm × 3.4 mm; 512 A-scans × 512 positions × 4 scans/position). Doppler OCT images were acquired with highly overlapped A-scans (~87% overlapping) from a relatively smaller area centered at the ONH (2 mm × 2 mm, 3000 A-scans × 200 B-scans). The camera image acquisition rate was set to 92 kHz, resulting in blood flow velocity detection range of [-17.4, 17.4] mm/s. A commercial ERG system (Diagosys LLC, Lowell, MA, USA) was integrated with the OCT system and the data acquisition for the two systems was synchronized. A custom visual stimulator was designed and integrated with the OCT imaging probe to allow for user choice of the intensity, duration and pattern of the visual stimuli[17,18]. During the OCT+ERG imaging procedure, light from the visual stimulator was focused at the pupil plane of the rat eye in order to generate a wide angle, almost uniform Maxwellian illumination of the retina. For this study, five scotopic ERG traces, separated by 3-minute dark period, were recorded with white light, single flash stimuli of 1 ms duration and five different illumination levels (-0.79~1.46 log scotopic cd·s/m^2^).

### Animal preparation and IOP elevation protocol

Eleven-week-old, male Brown Norway rats (~300g) were used in this study (n = 6). All experiments described here were approved by the University of Waterloo Animal Research Ethics Committee and adhered to the ARVO statement for use of animals in ophthalmic and vision research. Prior to the experiment, the rats were dark-adapted for at least 12 hours and the OCT+ERG data acquisition was carried out in a scotopic environment (631nm, <0.9 lux). The rats were anesthetized with isoflurane and oxygen mixture maintained at 1.5–2.5% throughout the experimental procedures. Afterwards, the rats were placed in a custom stereotaxic animal holder that allowed for XYZ and angular adjustment of the animal eye with respect to the stationary OCT+ERG imaging probe. Furthermore, the animal holder was designed for 360° rotation in a plane perpendicular to the OCT imaging beam, which allowed for easy switch between the left and right eye under the OCT+ERG imaging probe. The base of the animal holder was lined with a thermal pad (Kent Scientific, Torrington, CT, USA) in order to keep the rat body temperature between 36° and 38°C. One drop of 0.5% proparacaine hydrochloride (Alcaine, Alcon, Mississauga, ON, Canada) was applied to each eye, followed by one drop of pupillary dilator (0.5% tropicamide, Alcon, Mississauga, Canada). Topical anesthetic was also applied frequently to reduce the corneal sensitivity to the vascular loop (0.5% proparacaine hydrochloride, Alcaine; Alcon, Mississauga, ON), and artificial tears were used frequently (~ every 5 minutes) to hydrate the cornea to preserve its optimal optical clarity for the OCT imaging and improve the impedance of the ERG corneal electrode. The positive ERG electrode in the form of loop with 4 mm diameter, was gently placed on the rat cornea to avoid any IOP fluctuations. The negative ERG electrode was placed under the skin, behind the ear and the ground electrode was placed into the scalp between the ears. Vital signs, such as respiration rates, heart rates and body temperature were monitored and recorded every 10 minutes over the experiment.

An adjustable vascular loop (Sentinal Loops; Sherwood-Davis and Geck, St. Louis, MO, United States) was placed anterior to the equator of the eyeball of the right eye to provide controllable IOP elevations, while the left eye served as a contralateral control. Topical anesthesia (0.5% proparacaine hydrochloride, Alcaine, Alcon, Mississauga, Canada) was applied onto the cornea to reduce the rats’ sensation to the vascular loop. Different levels of elevated IOP were achieved by manually adjusting the tightness of the vascular loop, and the IOP was measured with a pre-calibrated corneal rebound tonometer (TonoLab, Finland). The IOP was raised unilaterally in steps from baseline (~10 mmHg) to 30 mmHg, 50 mmHg, 60 mmHg and 70 mmHg and then normalized by loop removal. At each IOP level, the rats were stabilized for ~10 minutes and the IOP was measured 3 times prior to the monocular OCT+ERG recordings.

### OCT imaging of the whole eye

Because the vascular loop introduces mechanical deformation of the eye ball that can cause changes in the axial length of the eye and corneal curvature, and therefore affect the amplitudes of the ERG a-wave, b-wave and oscillatory potentials (OPs), we used a swept source OCT system (SS-OCT) with sufficiently long scanning range (7 mm in free space) to evaluate those changes. Since the SS-OCT system was originally designed for imaging of the human cornea, its optical design and performance were described in an earlier publication from our group[29]. Briefly, the SS-OCT system utilizes a tunable laser (Axsun Technologies, Inc.) with sweep range centered at 1040 nm, sweep rate of 100 kHz and 50% duty cycle. It provides 7 μm axial and 15 μm lateral resolution and imaging range of 7 mm in free space. To evaluate changes in the shape of the rat eye during IOP elevation to levels ranging from 10 mmHg to 70 mmHg, SS-OCT volumetric scans were acquired from a 7 mm × 7 mm area centered at the corneal apex (700 A-scans × 700 B-scans). Because the optical length of the rat eye is larger than 7 mm, the rat eye images were wrapped around the SS-OCT zero delay line so that for each measurement the corneal apex overlapped with the retinal pigmented epithelium (RPE) layer of the retina, which provided sufficient contrast to allow for precise alignment. The axial eye length was computed as 2x the distance from the top edge of the SS-OCT image (corresponding to the zero delay line) and the location of the corneal apex. Changes in the corneal curvature were evaluated by measuring the height of the anterior chamber (distance between the pupil plane and the corneal apex) and the anterior chamber angle.

### Data analysis

Total retinal blood flow (TRBF) was evaluated using the Doppler angle irrelevant *en-face* method proposed by Srinivasan et al[30,31]. The absolute blood flow of each blood vessel was calculated by integrating the axial blood flow rate over the blood vessel area determined from the selected *en-face* image. TRBF of the retina was calculated as an average of the absolute total venous and arterial flow around the ONH. The procedure utilized for quantification of the microvascular density in different retinal layer involved three steps. First, the morphological OCT images were flattened and the flattening index was used to flatten the corresponding OCTA images. Then, all the flattened OCTA images were assembled into a 3D stack. Three major retinal layers, the nerve fiber layer and ganglion cell layer complex (NFL+GCL), the inner plexiform layer (IPL) and the outer plexiform layer (OPL) were segmented manually from the OCTA images. Second, maximum intensity projection was used to generate microvascular maps for each of these three layers. Third, to quantify the vascular density, the microvascular maps were Frangi filtered[32] and binary filtered using MATLAB’s Otsu’s threshold function[33]. Capillary density was calculated as the number of bright pixels in each layer. Note that the microvascular densities for the IPL and OPL were calculated excluding any areas with shadowing artifacts from major blood vessels (>35 μm) on the retinal surface.

The ERG traces were analyzed in terms of three metrics: a-wave, b-wave, and OPs, which represents mainly the response of photoreceptors, ON bipolar cells and Amacrine cells, respectively. As such, the ERG trace is decomposed as following. The ERG traces were fitted with a delayed Gaussian function to predict integrity of the phototransductive cascade[34] (PIII; MATLAB, MathWorks, MA, USA), and the amplitude the Gaussian function is considered as the a-wave amplitude. Next, this PIII portion is subtracted from the ERG trace, and ON bipolar cell response (PII) and OPs were separated by a Fourier filter (OPs: 75–300 Hz). Amplitude of the b-wave is computed as the maximum amplitude of the response from the brightest flash (1.46 log scotopic cd·s/m^2^). OPs were calculated as the difference of the root mean square (RMS) within a time window from t = 20 ms to t = 70 ms post flash and RMS of the background (t = -50 ms to t = 0). Because mechanical stress from the vascular loop at elevated IOP levels changed both the axial eye length and the corneal curvature of the rat eye, it also altered the coupling of the visual stimulus light into the eye and correspondingly, the illuminated area at the retina surface. Since the amplitude of the photoreceptor response is directly proportional to the number of stimulated photoreceptors, it is therefore dependent on the illumination spot at the retina. As we are interested in evaluating physiological changes in the retina associated with the IOP elevation and separating those from any changes induced by the increase in axial eye length that may affect the ERG metrics, in our study we discussed both the raw, unscaled versions of the ON bipolar cell response amplitude and OPs, as well as their normalized versions by the photoreceptor response.

### Statistical analysis

One-way repeated-measures ANOVA was used to determine differences in normalized TRBF as a function of IOP elevations, and two-way repeated-measures ANOVA was used to determine differences in retinal microvascular densities, ERG metrics, as a function of IOP and eyes. Greenhouse-Geisser corrections were used for epsilon values less than or equal to 0.75. Bonferroni-corrected multiple comparison tests were used post hoc to determine any differences between the loop conditions. p<0.05 is considered as significant difference.

## Results

### Morphological changes

**Fig 1:**
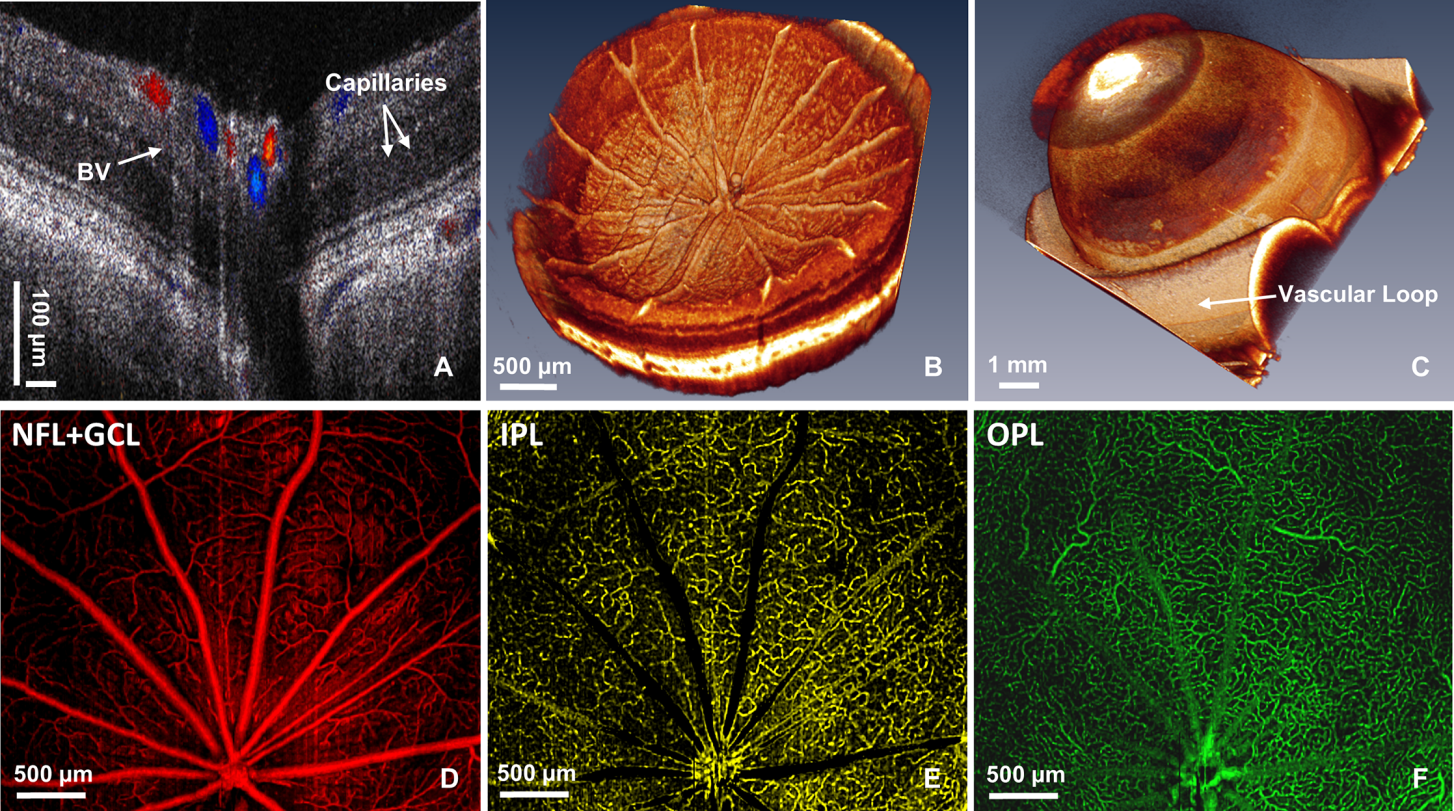
Representative morphological OCT, Doppler OCT and OCTA images of the rat eye. (A) Cross-sectional morphological OCT image of the rat retina overlaid with Doppler OCT to show the retinal vasculature with arteries marked in red and veins–in blue color. (B-C) Volumetric 3D OCT images of the rat retina and anterior eye chamber respectively. Vascular loop is placed anterior to the eye chamber (white arrow). (D-F) Examples of OCTA images from the NFL+GCL, IPL, and OPL layers respectively.

Fig 1A shows a morphological OCT image overlaid with its corresponding Doppler OCT image for normal IOP. Surface blood vessels (BV) are color coded, where blue color represents veins and red color represents arteries. Capillaries appear as white dots in the inner retina (white arrows), and bidirectional blood flow is also observed in large choroidal blood vessels. A wide angle 3D image of a healthy rat retina is shown in Fig 1B, while Fig 1C shows a presentative 3D image of the anterior rat eye with a vascular loop. Fig 1D–1F show the retinal microvasculature for the three segmented retinal layers: NFL+GCL, IPL, and OPL, coded in red, yellow, and green colors respectively.

**Fig 2:**
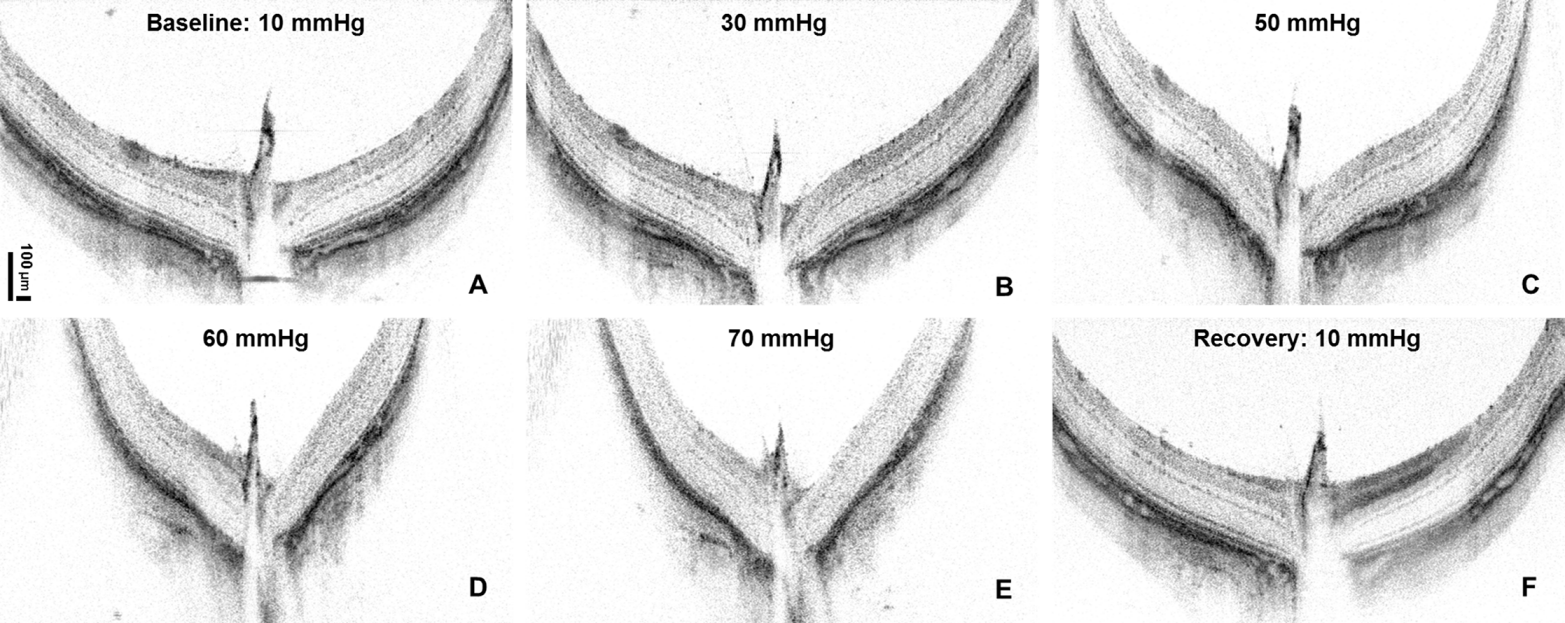
Representative cross-sectional OCT images of the ONH showed progressive deformation of the ONH associated with elevated IOP (A-E). (F) ONH morphology recovered 30 minutes after loop removal.

Fig 2 shows a series of cross-sectional morphological OCT images acquired through the center of the ONH for different levels of the IOP. The step-wise IOP elevation caused progressive deformation of the ONH. The mechanical deformation of the rat eye ball also caused a focal shift of the OCT imaging beam from the surface of the retina toward the posterior retina which is noticeable for IOPs > 50 mmHg or higher, resulting in lower contrast of the retinal images. Within 30 minutes from removal of the vascular loop, the ONH recovered to its original shape, however, whether there is a permanent damage associated with acute IOP elevation needs to be examined by histology.

### Axial eye length changes

**Fig 3:**
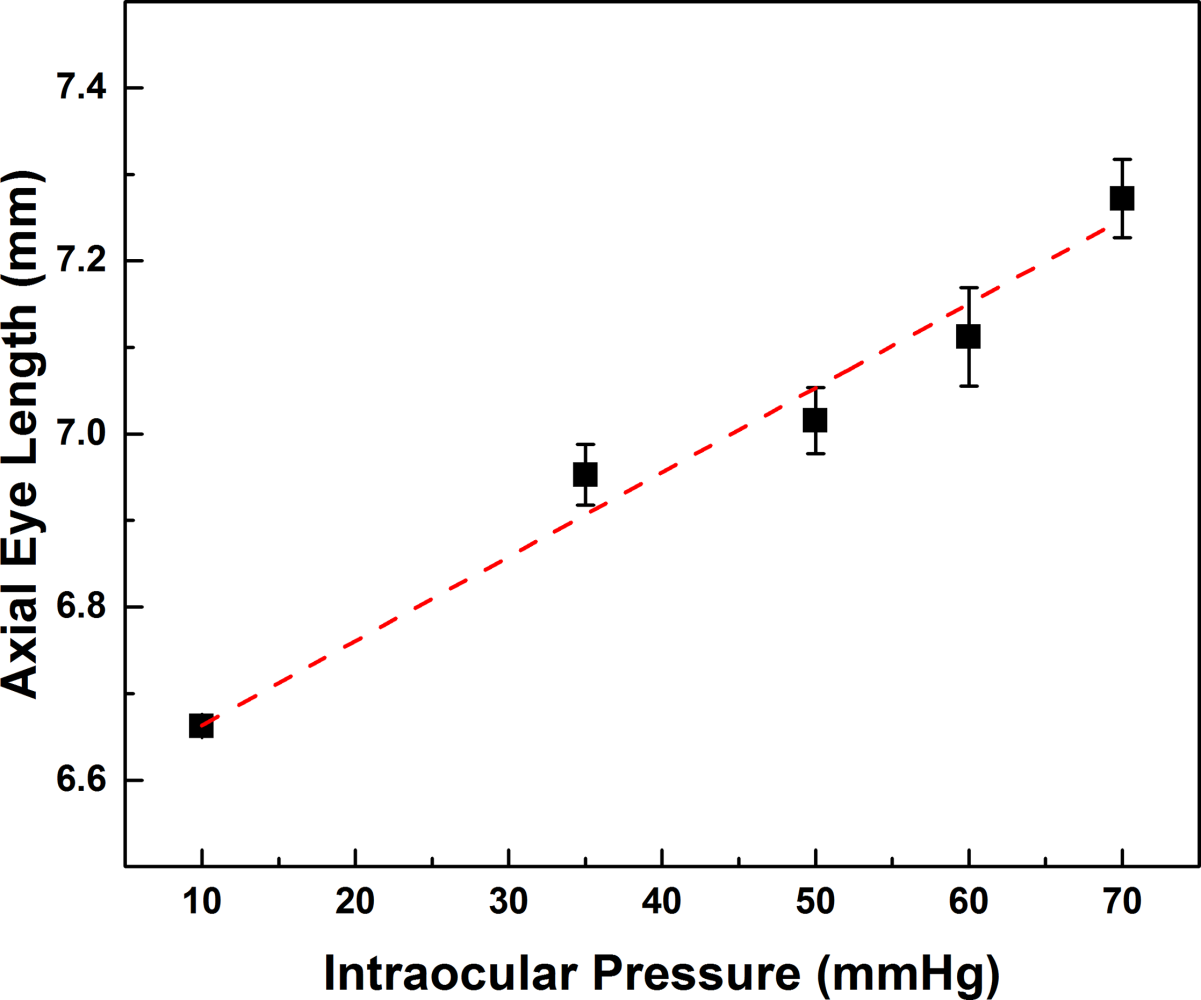
Correlation between IOP and axial eye length. Linear increase of the axial eye length is detected with IOP increase (red dashed line, R square = 0.91). Data are presented as Mean ± S.E.

Analysis of the SS-OCT data showed IOP associated changes in the shape of the rat eyeball. Fig 3 demonstrates the relationship between IOP and the axial eye length. A linear fit of the data shows a strong correlation between the IOP and the axial eye length (red dashed line, r-square = 0.95). Further analysis of the SS-OCT images showed no significant changes of the anterior eye chamber depth or the anterior angle, suggesting that there were negligible changes to the curvature of the rat’s cornea.

### TRBF changes

**Fig 4:**
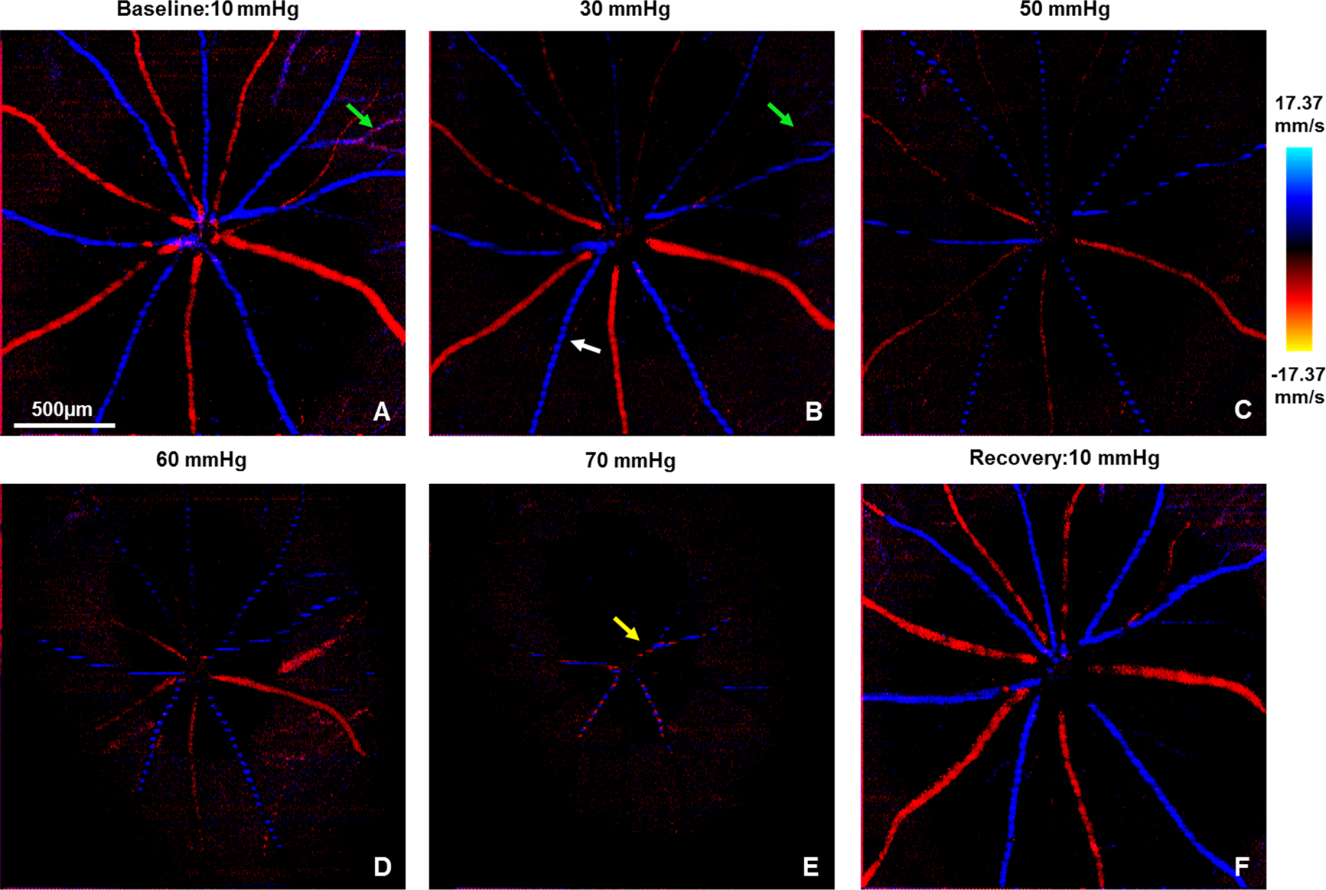
Representative *en-face* max projected Doppler OCT images in response to different IOP levels. Blue and red color represent arteries and veins, respectively. White arrow indicates the pronounced cardiac pulsation on the retinal arterial flow. Yellow arrow indicates that the cardiac pulsation caused retinal flow direction change at highly ischemic level IOP. Green arrows: vanished visualization of small vessels at 30 mmHg IOP. Scale bar = 100μm.

Fig 4 shows maximum projection Doppler OCT images of the retinal blood flow at the ONH and its vicinity. Blue and red color correspond to arteries and veins respectively. The green arrows in Fig 4A and 4B indicate the apparent loss of blood flow in a small vessel for IOP of 30 mmHg, without apparent significant alteration of the blood flow in the larger retinal blood vessels. When the IOP was elevated to 50 mmHg and higher, significant reduction of the retinal blood flow was observed, and the blood flow in the peripheral area was affected more strongly compared to the ONH as shown in Fig 4D and 4E. Pulsation of the retinal arteries associated with the cardiac cycle can be observed as dark lines across the blue colored arteries in all images in Fig 4. For IOP elevation of 70 mmHg, the pulsation in the retinal arteries even caused an apparent retinal blood flow directional change, observed as change between blue and red color in Fig 4E (yellow arrow). The same effect was also observed by Zhi et al.[21] in a retinal blood flow study, where an anterior chamber cannulation method was used to elevate the IOP in rats.

**Fig 5:**
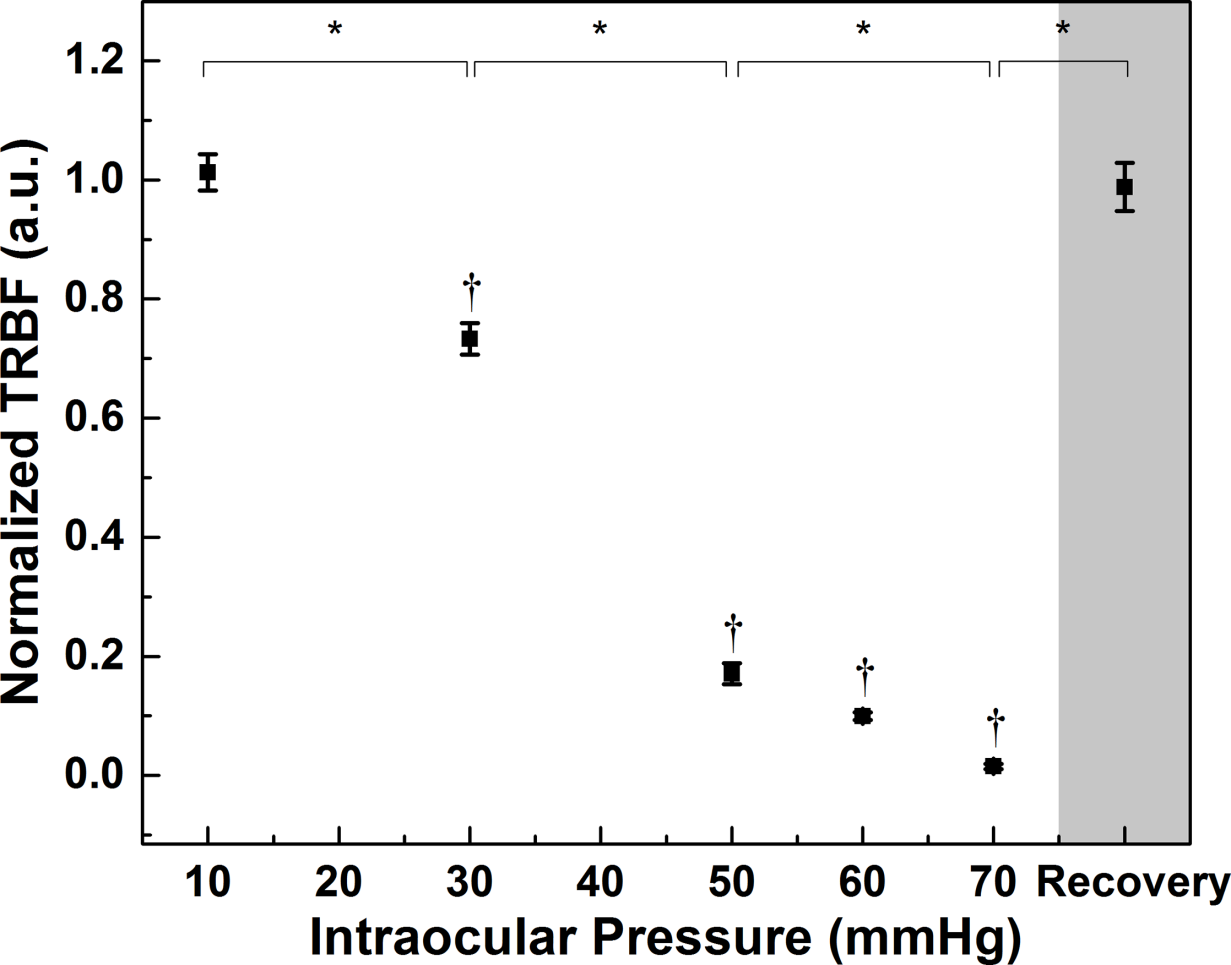
Effect of IOP on TRBF (Mean ± SE). Progressive decrease of TBRF is associated with increasing IOP from 10 mmHg to 70 mmHg, while full recovery was observed 30 min post loop removal. Data are presented as Mean ± S.E., normalized to the untreated eye. †Significant difference compared to baseline (p<0.01). *Significant difference between different IOP steps (p<0.01).

Statistical results for the TRBF measured for different levels of IOP are shown in Fig 5. The TRBF change measured in the treated eye was normalized to the untreated eye, as the existence of large variance of retinal TRBF among animals[35, 36] is well known. Normalized TRBF was significantly lower at IOP of 30 mmHg (p<0.01) and decreased monotonically with higher IOP. Specifically, TRBF at 30 mmHg and 50 mmHg IOP had a steep decrease from baseline and was at about 70% and 20% of baseline compared to the untreated eye (p <0.01). When the IOP was raised above 50 mmHg, the decrease gradient was smaller, though the change in the TRBF between those three data points was not significant (p = 1.00). Note that the blood flow at 70 mmHg is close to the detection threshold of our OCT system due to the significantly altered geometry of the eyeball at that IOP level. The TRBF measured during the recovery phase (30 min after removal of the vascular loop), had a significant increase relative to the measurement at 70 mmHg (p<0.01) and fully recovered to the pre-loop level (p = 1.00).

### Blood perfusion changes

**Fig 6:**
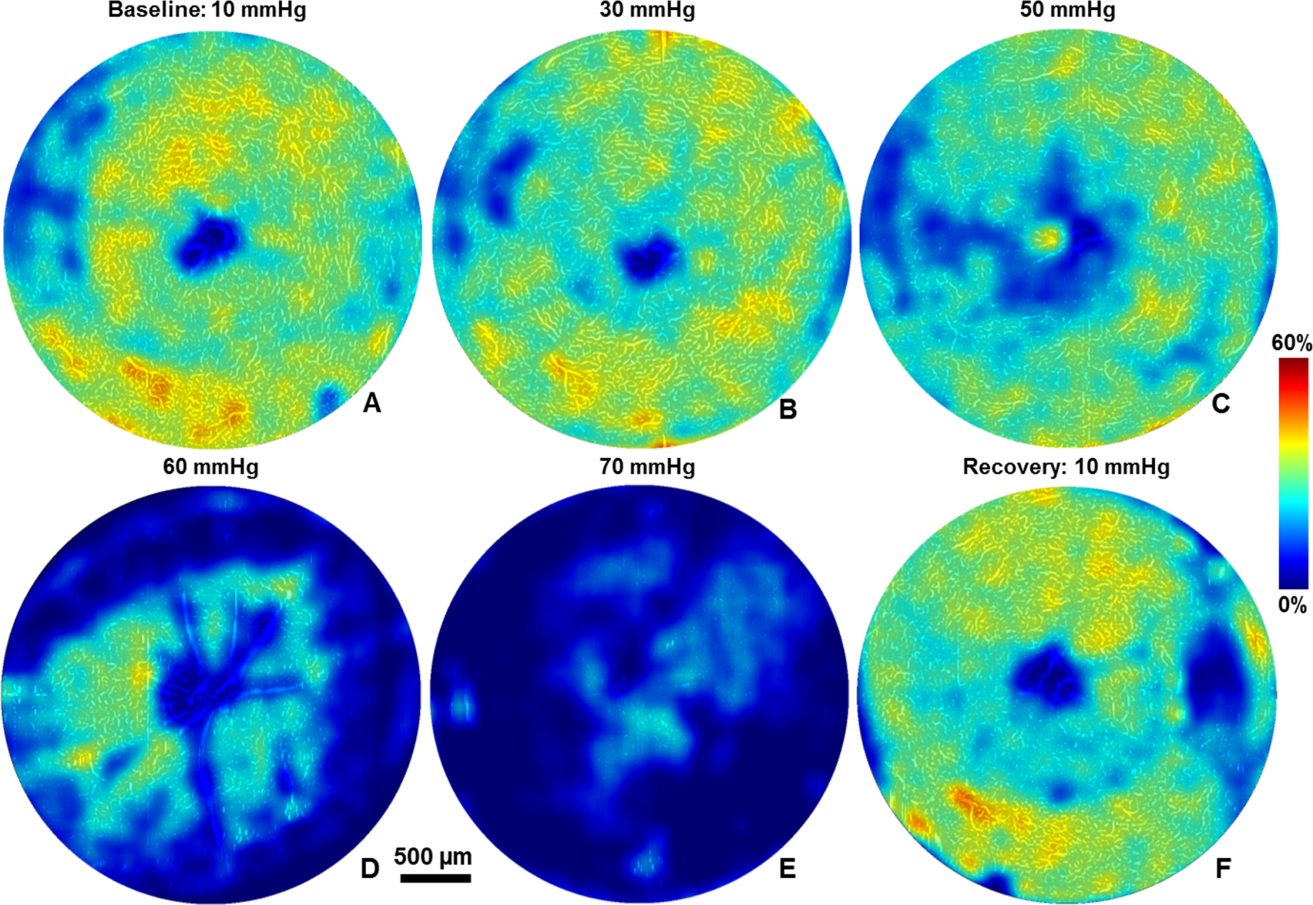
Representative microvascular maps (white) overlaid with density maps (jet color) in OPL at different IOP levels. Different colors denote the microvascular density from 0% to 60%.

**Fig 7:**
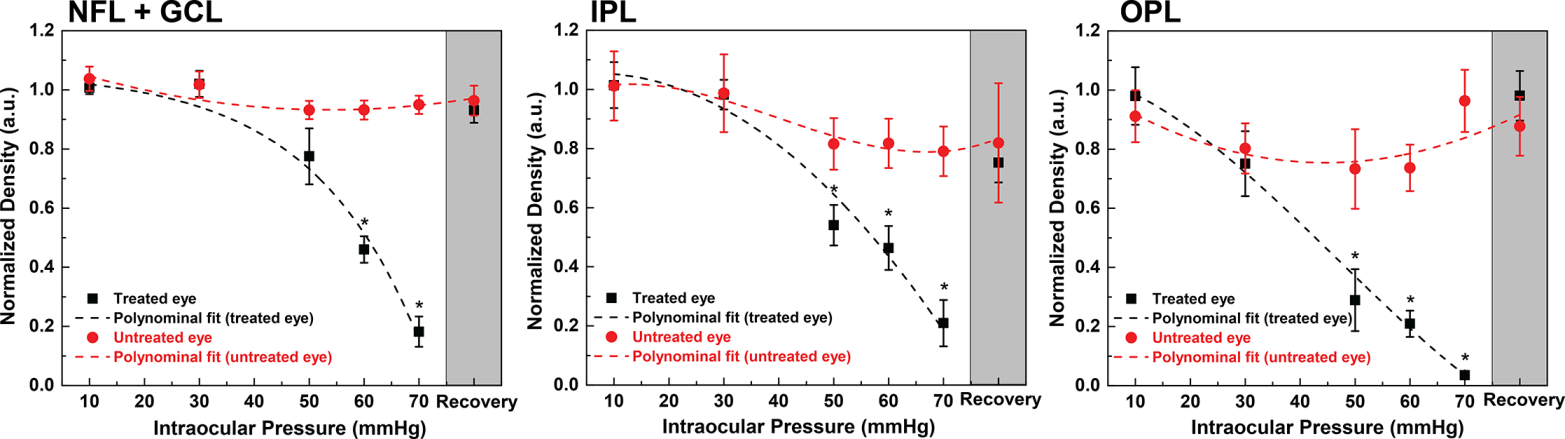
Quantification of microvascular density in NFL+GCL, IPL, and OPL at different IOP levels. Density data in both eye are represented in Mean ± S.E. with fitted polynomial function (n = 3). Recovery data in the treated eye is excluded from fitting. *Significant difference between two eyes (p<0.05).

Representative retinal blood perfusion maps for the OPL, generated for normal and elevated IOP are shown in Fig 6. For better visualization of the IOP induced changes, the microvascular maps were overlaid with their corresponding blood vessel density maps. Shadow artefacts from the surface retinal blood vessels were filtered out by excluding the blood vessels with size lager than 35 μm. Statistical results summarizing the retinal blood perfusion changes in the three segmented retinal layers (NFL+GCL, IPL and OPL) associated with the IOP elevation are shown in Fig 7. The microvascular density was normalized to the averaged treated eye value for the baseline IOP. Overall, in all three layers, the microvascular densities in the treated eye changed significantly with the elevated IOP (all p<0.05). Although the microvascular density in the untreated eye remained unchanged in all three layers for normal and elevated IOP (p = 1.00), it is important to note that the microvascular density decrease trend observed in the GCL+NFL and OPL, is most likely due to fellow eye effect, prolonged anesthesia or possible progressive dehydration of the corneas between successive application of artificial tears, which is our case was done every few minutes. In the treated eye, in all three layers, the microvascular density was significantly lower than the untreated eye when the IOP was raised to 60 mmHg or 70 mmHg (p<0.05). When the IOP was raised to 50mmHg, significant interocular differences were only detected in IPL and OPL. Thirty minutes after loop removal, recovery of microvascular density to the baseline in all three retinal layers was observed (p = 1.00).

### Functional changes

**Fig 8:**
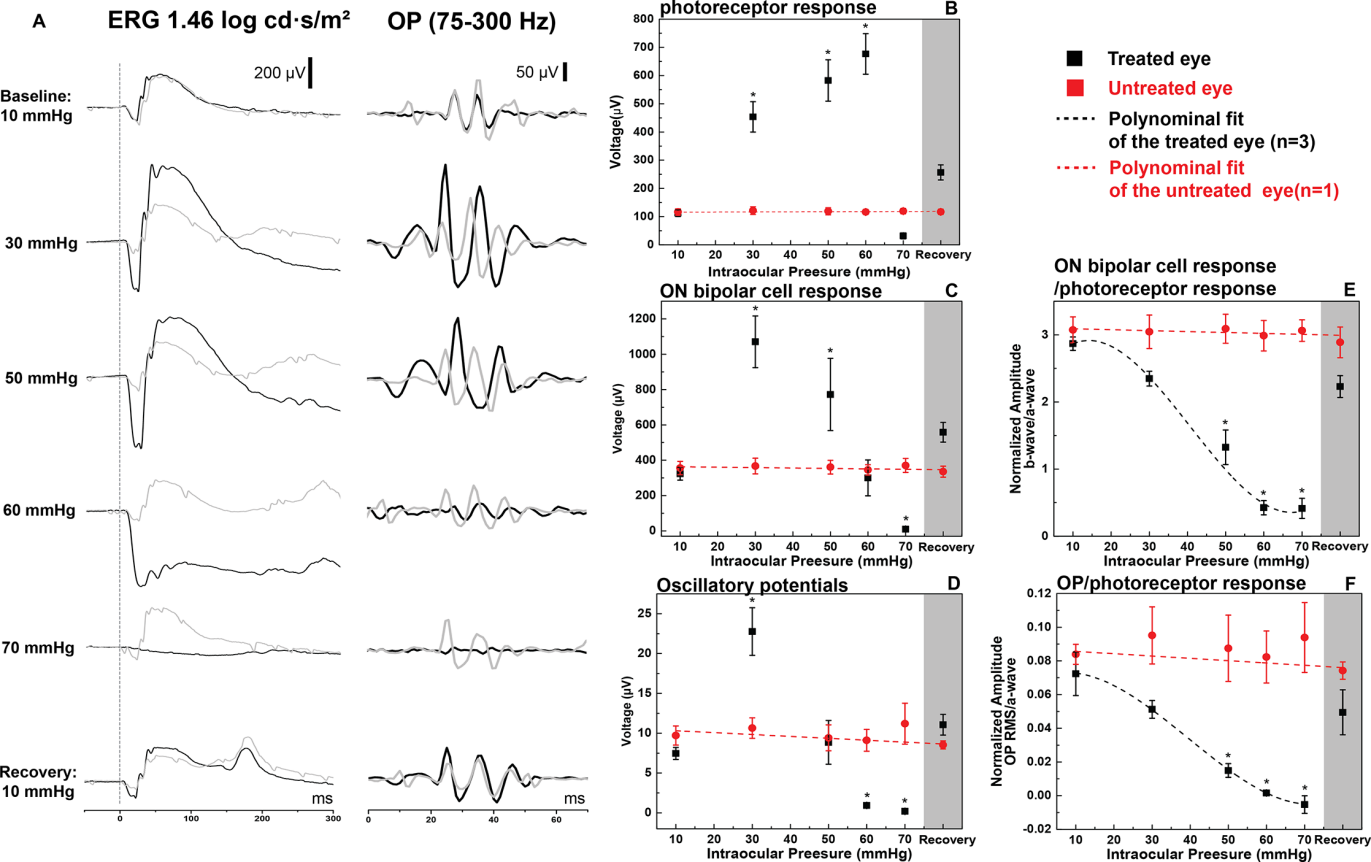
(A) Representative ERG traces and the corresponding OPs at different IOP levels. Dashed line labels the onset of the visual stimulation. (B-D) Statistics of photoreceptor response, ON bipolar cell response and OPs at different IOP levels. (E and F) Normalized ON bipolar cell response and OPs related to the photoreceptor response, respectively. *Significant difference between two eyes (p<0.05). Data are presented as: Mean ± S.E.

Representative ERG traces and the extracted OPs for different IOP elevation levels, acquired from one animal are presented in Fig 8A. Black and grey color correspond to the treated eye and the untreated eye respectively, and the dashed line indicates the onset of the visual stimulation. The untreated eye response exhibited good stability during the loop-on procedure, indicating lack of fellow-eye effect for elevated IOP. Fig 8B–8D summarize statistical data for the ERG metrics (photoreceptor response, ON bipolar cell response and OPs) for a group of 6 rats. The a-wave amplitudes increased significantly from baseline to 60 mmHg (p<0.01), and there was significant difference between the data acquired at 30 mmHg and 60 mmHg in the treated eye (p<0.01). When the IOP was elevated to 70 mmHg, the a-wave amplitudes measured in the treated eye were on average lower than those in the untreated eye (p = 1.00). The ERG b-wave amplitudes behaved in a very different manner. They increased significantly from baseline and peaked for IOP of 30 mmHg (p<0.01), then progressively declined to the same value measured in the untreated eye at ~60 mmHg. For IOP at 70 mmHg, the b-wave magnitude was lower than the untreated eye (p = 0.03). Similar to the ERG b-wave amplitudes, OPs RMS increased at IOP of 30mmHg (p<0.01), and returned to baseline when the IOP reached 50 mmHg (p = 1.00). Afterward, it continued to decrease to a level significantly lower than the untreated for IOP of 60 mmHg and 70 mmHg (both p<0.01). The normalized ERG b-wave amplitudes and the OPs RMS relative to a-wave amplitudes are presented in Fig 8E and 8F respectively. In contrast to the unscaled versions, the normalized b-wave amplitudes and the OPs RMS decreased monotonically with the IOP elevations, and for IOP levels between 50 mmHg to 70 mmHg, both the normalized b-wave amplitudes and the OPs RMS were significantly lower than the ones measured from the untreated eye (p<0.01 in all comparisons), while no significant intraocular difference was detected at 30 mmHg (b-wave: p = 0.10; OP: p = 0.59). After loop removal, the a-wave amplitudes, b-wave amplitudes and OPs RMS recovered to a value higher than the ones in the untreated eyes, while the normalized b-wave amplitudes and OPs RMS recovered to values lower than ones in the untreated eyes. However, these differences were not significant (for all comparisons, p>0.05).

## Discussion

In this study, we applied a vascular loop anterior to the eye chamber in order to raise IOP, and measured simultaneously elevated IOP induced changes in the retinal morphology, blood flow/perfusion and function. The vascular loop method provides several advantages over other IOP elevation approaches: a) leaves clear aperture for the OCT imaging beam; b) provides controllable IOP elevation by simply tightening or loosening the vascular loop; c) it is an easy and inexpensive approach that doesn’t require specific expertise or additional instruments.

Morphological data from our study showed deformation of the ONH with elevated IOP, which increased progressively in magnitude with increase of the IOP level. Similar ONH morphological change with elevated IOP was observed in a number of studies that utilized different IOP elevation methods, including the vascular loop technique, as well as different IOP levels[25,37,38]. In our study, we also observed a recovery of the ONH to its original shape at about 30 min after loop removal even after an IOP insult to an ischemic level of 70 mmHg. This result suggests that if there is any early damage to the ONH tissue associated with a short term, acute elevation of IOP to ischemic levels, that damage is below the detection threshold of the OCT system and needed to be certified by other techniques, like histology and immunochemistry. Other groups have also shown that the ONH structure recovered to baseline immediately after acute IOP elevation to 100 mmHg[22], and 30 minutes after elevating IOP to 70 mmHg for 60 minutes[39], both of which used anterior chamber cannulation to raise IOP.

By cannulating the anterior chamber to elevate IOP[21], retinal blood flow was reported to decrease significantly when the IOP was raised to 30 mmHg, and the TRBF dropped to lower than 50% of baseline for IOP higher than 60 mmHg. Our results obtained with the vascular loop method showed somewhat different behavior of the TRBF: significant decrease in the TRBF was observed when the IOP was elevated to 30mmHg. However, for IOP of 50 mmHg, the TRBF was at ~20% of its baseline value or compared to the untreated eye. One possible explanation of this difference is that the vascular loop compressed the episcleral vein, and may have a similar effect on the retinal and choroidal flow as scleral buckling or encirclement. Scleral bucking and encirclement have been well documented to affect the retinal and choroidal blood flow[40–44]. Specifically, Ogasawara[44] and Regillo[42] reported up to 53% decrease of arterial flow velocity in patients after scleral buckling and circling procedures, and Sugawara[41] reported a sustained reduced choroidal flow from 2–4 weeks after sclera buckling. Therefore, compression of the ocular tissue with the vascular loop could affect the blood circulation in the eye and alter significantly retinal blood flow and choroidal blood flow.

Decrease in the capillary density of the IPL and OPL has been observed previously for IOP elevation to levels higher than 60 mmHg with the cannulation method[21]. In that study, microvascular density in the OPL measured at IOP of 60 mmHg was at 60% of the baseline value. In contrast, results from our study showed that with the vascular loop method, the OPL microvascular density for the same IOP level was at ~20% of baseline value. Since both studies were conducted on the same strain of rats, there are 3 major factors that can contribute to the discrepancy between results from the two studies. One factor is the method of IOP elevation. As mentioned above, similar to the scleral bucking and encirclement methods, the vascular loop may affect blood flow and perfusion in the retina more strongly than fluid pressure in the posterior chamber of the eye induced by anterior chamber cannulation. A second factor to consider is the difference in the OCT systems’ design and performance. Although both systems are SD-OCT and provide sensitivity of ~ 100dB, the one used by Wang’s group had an image acquisition rate of 240 kHz, while the image acquisition rate for the system used in our study was 92 kHz. The OCT systems’ design and image acquisition rate will affect the sensitivity of the OCTA method utilized by both research groups to detect microvasculature in the OPL layer. Lastly, the eye defocus resulted in OCT signal reduction, which could also contribute to the observed decrease in the microvascular density.

Measurements of the retinal function for a moderate IOP elevation level (IOP = 30 mmHg) and a highly ischemic IOP level (70 mmHg) agreed with results from a previous study with a vascular loop by Choh et al.[25] and also extended the observation of retinal function transition from normal through non-ischemic, to ischemic IOP levels. Shot-term moderate level IOP elevation (~35 mmHg) is associated with supranormal ERG amplitudes as well as supranormal positive scotopic threshold response (pSTR) amplitudes[25], which mainly reflects the function of retinal ganglion cells. One possibility is the extended eye length during acute IOP elevation, as also evidenced from the axial eye length measurements. However, Westall et al.[45] reported reduced rather than increased ERG amplitudes with longer eyes. Additionally, the increased illumination area during IOP elevation does not adhere to the 5X increase of the ERG amplitudes. Supranormal ERG can be explained with loss of retinal dopaminergic amacrine cells[46,47], blockage of retinal dopamine receptors[48,49], gestational low level lead exposure[50,51], loss of a mitochondrial ATP transporter in Ant1-/- mice[52] and NO injection[53], which was discussed in more details in the previous publication[25].

The ratio between the b-wave amplitude and the a-wave amplitude considers photoreceptor’s activity as an input and post-synaptic neuronal activity as an output[54]. The ERG b-wave/a-wave amplitude ratio has been proven to serve as a good indicator of diseases caused by retinal ischemia[55], such as central retinal vein occlusion[56,57] and retinal artery occlusion[58]. Moreover, Kong and his colleagues[59] used cannulation to raise the IOP acutely in rats, and reported reduction of all three ERG metrics (a-wave, b-wave amplitudes and the OPs RMS) with IOP elevations, where the gradients of the b-wave amplitudes and OPs RMS reduction were larger than that of the a-wave amplitudes. Furthermore, OPs are also sensitive to retinal ischemia[54]. Sperous[60] showed attenuated OPs with mild retinal ischemia and OPs were also regarded as indicator of background retinopathy[61,62]. In our study, the normalized b-wave amplitudes and OPs RMS showed the similar trend to the measured TRBF as a function of IOP, and thus could serve as more sensitive markers to IOP-associated retinal ischemia than the unscaled ones.

Half an hour of post-loop phase is not sufficient for complete recovery of the retinal function to baseline in this study. Specially, at the end of the 30 min recovery phase, the all ERG metrics were still higher than their corresponding baseline values. Similarly, the normalized b-wave amplitude and the OPs RMS in the treated eye were still ~35% and ~50% lower compared to baseline and to the untreated eye. He et al.[63] found that the recovery time for retinal function is dependent on the peak IOP level. He et al.[63] elevated the IOP to 70 mmHg for 30 minutes by anterior chamber cannulation and observed that retinal function, evaluated by negative scotopic threshold responses (nSTR), and recovered only to 50% of its baseline value for a recovery time of 30 minutes. In contrast, retinal blood flow and blood perfusion recovered within 30 minutes, which is consistent with several previous studies[21–23,64,65]. The recovery rate difference between retinal blood flow / blood perfusion and retinal function in response to acute IOP elevations indicates different recovery mechanisms.

One limitation of this study is lack of the retinal ganglion cell (RGC) function measurement. Indeed, RGC is sensitive to acute IOP elevation[66], and RGC function is usually assessed by other techniques, including scotopic threshold response[66]. STR measurements requires low stimulating intensities to isolate the RGC function, and the LED stimulator in our system cannot achieve such low intensities with confident intensity calibration. Further study with modified stimulator, e.g. handheld ganzfeld, will help to measure the retinal blood flow/perfusion and the RGC function simultaneously.

In summary, we used a combined OCT + ERG system to measure simultaneously changes in the retinal structure, function, blood flow and blood perfusion in response to acute IOP elevation to non-ischemic and ischemic levels in rats. Nonlinear ERG responses were detected as a function of IOP, and the normalized b-wave amplitudes and OPs RMS showed better correlation to the retinal ischemia. During the recovery phase, retinal blood flow/perfusion recovered to its baseline value after 30 min from loop removal, while retinal function did not recover fully within 30 min of loop removal. These results show the difference of retinal function and blood flow/blood perfusion in response to acute IOP elevation and recovery. Further studies including establishing mathematical model and modifying stimulator for STR measurement will improve our understanding of the relation between retinal function and blood flow/perfusion.
